# Sleep and psychiatric disorders: Bidirectional interactions and shared neurobiological mechanisms

**DOI:** 10.1371/journal.pmen.0000531

**Published:** 2025-12-31

**Authors:** Anna Hyndych, Kateryna Koval, Natalia Dzeruzhynska, Edward C. Mader

**Affiliations:** 1 Sleep and Health Research Program, Department of Psychiatry, University of Arizona College of Medicine, Tucson, Arizona, United States of America; 2 Department of Psychiatry and Narcology, Bogomolets National Medical University, Kyiv, Ukraine; 3 Department of Neurology, Louisiana State University Health Sciences Center, New Orleans, Louisiana, United States of America; PLOS: Public Library of Science, UNITED KINGDOM OF GREAT BRITAIN AND NORTHERN IRELAND

## Abstract

Sleep is critical for emotional regulation, memory, and cognitive performance. Sleep disturbances, including insomnia, hypersomnia, and circadian misalignment, are highly prevalent and clinically significant across various psychiatric disorders. Once considered secondary, sleep problems are now recognized as active contributors to the onset, course, and relapse of mental illness. This narrative review synthesizes current evidence on the bidirectional interactions between sleep and major psychiatric conditions such as major depressive disorder, bipolar disorder, anxiety disorders, posttraumatic stress disorder, schizophrenia, attention deficit and hyperactivity disorder, and substance use disorders. We highlight convergent neurobiological mechanisms, including dysregulation of circadian systems, neurotransmitter networks (GABA, serotonin, dopamine, orexin), affective circuitry (prefrontal-amygdala interactions), and stress-immune pathways. Findings consistently show that sleep problems are transdiagnostic features, impacting diagnostic presentation, prognostic trajectories, and underlying pathology. For instance, chronic insomnia increases depression risk, sleep loss can precipitate manic episodes, and distinct sleep architecture anomalies are linked to schizophrenia. Sleep disturbances also predict worse outcomes in substance use disorders, including increased craving and relapse risk. Sleep is a tractable factor in mental health, offering a potent intervention leverage point. Routine, structured sleep assessment should be integrated into psychiatric care, emphasizing first-line behavioral and chronobiological strategies like Cognitive Behavioral Therapy for Insomnia (CBT-I) and light/rhythm therapies. Directly addressing sleep significantly improves psychiatric outcomes, reducing symptoms of depression and anxiety, decreasing suicidal ideation, and lowering relapse risk in bipolar disorder and psychoses. Future research should prioritize causal designs, mechanistic neuroimaging, biomarker identification, and responsible integration of objective measurement technologies and artificial intelligence for early warning systems and personalized treatment protocols.

## 1. Introduction

**Fig 1:**
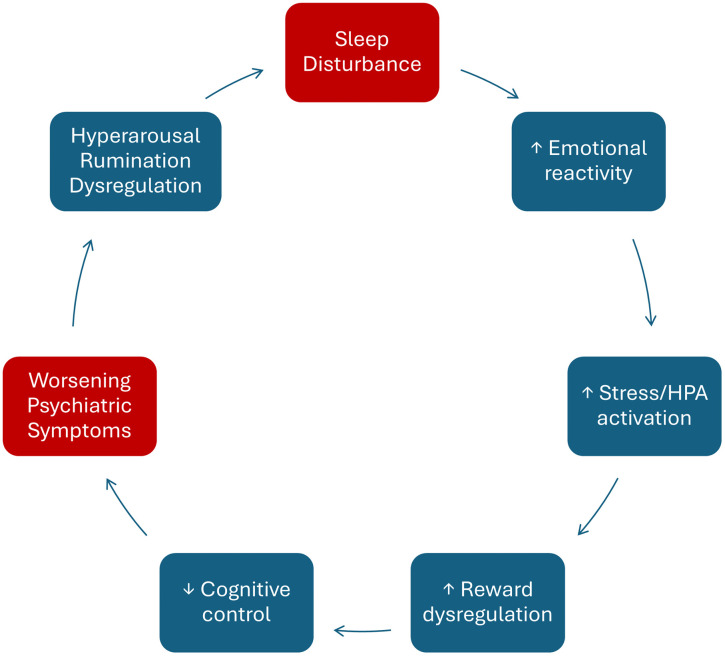
Bidirectional cycle linking sleep disturbance and psychiatric symptoms. Conceptual diagram illustrating how sleep disturbance triggers increases in emotional reactivity, stress and HPA-axis activation, reward-system dysregulation, and reductions in cognitive control. These changes contribute to worsening psychiatric symptoms, which in turn maintain hyperarousal, rumination, and regulatory disruption, perpetuating the cycle. *HPA = hypothalamic–pituitary–adrenal.*

Sleep is a fundamental biological process that supports emotional regulation, memory, and cognitive performance [1,2]. Across psychiatry, convergent evidence shows that sleep disturbances: insomnia, hypersomnia, and circadian misalignment, are highly prevalent and clinically consequential, cutting across diagnostic boundaries and associating with worse symptoms and functioning [3–5]. Historically framed as secondary to mental illness, sleep problems are now recognized as active contributors to the onset, course, and relapse of a psychiatric disorder [3]. Longitudinal and cohort data indicate that insomnia prospectively increases risk for depression and broader psychopathology [6–9], predicts psychotic-like experiences in youth [10], and forecasts internalizing symptoms in population cohorts [11]. Genetic causal inference further supports bidirectionality between sleep traits and psychiatric risk [12,13]. Concurrently, clinical reports and reviews highlight how psychiatric symptoms, such as rumination, hyperarousal, and anhedonia, hinder sleep regulation, forming self-perpetuating cycles that sustain the illness [3,14] (Fig 1).

From a mechanistic perspective, common biological pathways likely link sleep dysregulation and psychopathology. Circadian rhythm disruption is a recurring theme, with animal and human studies pointing to dysfunction of the suprachiasmatic nucleus, variations in clock genes, and network-level failure in anxiety, mood, and psychotic disorders [15–19]. Neurochemical systems central to sleep: serotonin, dopamine, gamma-aminobutyric acid (GABA), and related modulators, also play transdiagnostic roles in affect, salience, and cognitive control, providing substrates for reciprocal sleep-symptom effects [20,21]. Affective neuroscience adds a complementary line: rapid eye movement (REM) and sleep loss alter amygdala-prefrontal dynamics and emotional memory processes needed for fear resolution and mood regulation [22,23], and inflammatory axes connect chronic sleep disturbance with elevated cortisol responses and low-grade inflammation seen in multiple disorders [24–28] and psychopathology share interconnected circuitry rather than intersecting only incidentally [3,19,29].

This narrative review synthesizes current evidence on these reciprocal relationships across major depressive disorder, bipolar disorder, anxiety disorders, posttraumatic stress disorder (PTSD), schizophrenia, attention deficit and hyperactivity disorder (ADHD), and substance use disorders. We emphasize convergent neurobiology: circadian systems, neurotransmitter networks, affective circuitry, and stress-immune pathways, and derive clinical implications for integrated assessment and sleep-focused interventions in routine psychiatric care.

We consulted peer-reviewed literature indexed in PubMed/MEDLINE, PsycINFO, Scopus, and Google Scholar, along with practice guidelines from specialist organizations (e.g., AASM, FDA). Searches covered January 1990 through March 2025 and used combinations of sleep-related terms (e.g., *insomnia*, *sleep disturbance*, *REM sleep*, *circadian rhythm*), psychiatric disorder terms (*depression*, *bipolar disorder*, *anxiety*, *PTSD*, *schizophrenia*, *ADHD*, *autism spectrum disorder*, *substance use*), mechanistic terms (*HPA axis*, *inflammation*, *orexin*, *neurocircuitry*), and treatment-related terms (*CBT-I*, *chronotherapy*, *IPSRT*, *melatonin*, *orexin antagonists*). Boolean operators (AND/OR) were used to refine queries, and reference lists of key articles were reviewed to identify additional sources.

Although this is a narrative review, we aimed to differentiate areas where the evidence base is relatively strong from domains supported by more preliminary findings. Where available, we give greater weight to randomized controlled trials, meta-analyses, and large prospective cohort studies, which provide more robust support for causal or temporal associations. In contrast, findings generated from small laboratory experiments, cross-sectional clinical samples, or uncontrolled designs are described as tentative or hypothesis-generating. Many studies in the sleep–psychiatry literature rely heavily on self-report measures, short follow-up periods, or heterogeneous samples; therefore, some associations should be interpreted cautiously. When relevant, we explicitly note when conclusions rest on stronger versus weaker methodological foundations.

## 2. Sleep disturbances as a transdiagnostic feature of psychiatric illness

Sleep symptoms cut across diagnostic boundaries and carry diagnostic, prognostic, and mechanistic significance. In DSM-5, they are both primary (within the Sleep-Wake Disorders) and criterion-level features in major psychiatric categories: insomnia or hypersomnia in depressive episodes, decreased need for sleep in mania/hypomania, sleep disturbance in generalized anxiety, and insomnia/nightmares within trauma- and stressor-related disorders; in neurodevelopmental, neurocognitive, obsessive-compulsive, and eating disorders, they are commonly reported as associated features rather than defining criteria [30]. Framing sleep as a transdiagnostic construct clarifies shared pathways (arousal, circadian, immune) and highlights targets that generalize across conditions.

Prospective data show that sleep problems are not merely epiphenomenal. Chronic insomnia confers ~2–3 × risk for incident depression and predicts a more relapsing course; broader longitudinal syntheses link persistent insomnia to later anxiety and other disorders [7–9]. Beyond mood disorders, actigraphic disturbance in youth at clinical high risk for psychosis forecasts subsequent positive-symptom escalation [31], and in opioid use disorder, higher insomnia severity at intake and post-treatment predicts return to use and non-fatal overdose [32].

The reverse direction is likewise evident. Disorder-specific processes disrupt sleep initiation, depth, and continuity: rumination and worry drive nocturnal cognitive arousal in mood-anxiety disorders [33]; sleep loss amplifies affective reactivity and erodes cognitive control [34]; in PTSD, sleep is characterized by reduced efficiency and slow-wave sleep with increased wake after sleep onset, while REM alterations are variable, and trauma-related nightmares are common [35]; substance use maps onto the binge-withdrawal-preoccupation cycle with characteristic sleep deterioration [36]. In neurodevelopmental conditions ASD/ADHD), alterations in melatonin and neuromodulatory systems (GABA, norepinephrine (NE)/5-hydroxytryptamine (5-HT)/ dopamine/histamine) provide shared circuit substrates linking sleep and core symptomatology [37,38]. Together, these reciprocal pathways position sleep disturbance as both an upstream risk factor and a downstream amplifier of psychopathology.

## 3. Shared neurobiological and physiological substrates

**Fig 2:**
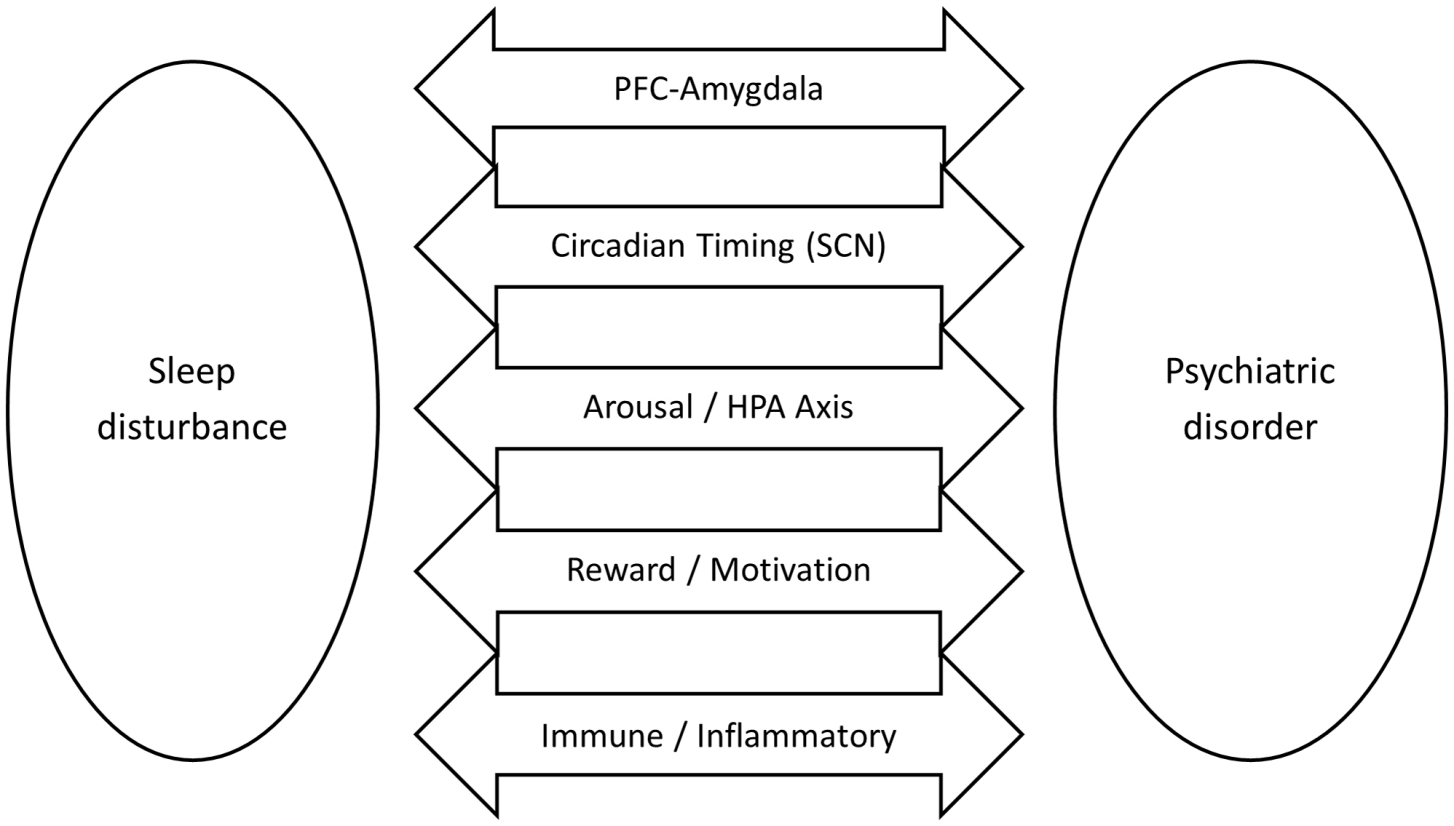
Transdiagnostic sleep–psychopathology mechanisms. Conceptual diagram illustrating shared pathways linking sleep disturbance to psychiatric disorders. Arrows indicate core mechanistic domains implicated across diagnoses, including PFC–amygdala circuitry, circadian timing regulated by the suprachiasmatic nucleus (SCN), arousal and HPA-axis activation, reward/motivation pathways, and immune–inflammatory signaling.

The high comorbidity between sleep disturbance and psychiatric illness reflects overlapping disruptions in brain circuitry, neuromodulators, circadian timing, and immune-endocrine function. Here, we highlight brain structures most consistently implicated in both sleep regulation and psychopathology (Fig 2).

### 3.1. Brain structures involved in sleep and psychopathology

#### Prefrontal cortex (PFC).

Acute sleep loss compromises prefrontal function. Neuroimaging meta-analysis and multimodal studies show reduced frontal recruitment and weakened fronto-cortical connectivity after a night of total sleep deprivation—patterns consistent with impaired top-down control [39,40]. Analogous prefrontal circuit abnormalities are prominent in clinical populations: in major depressive disorder, standardized fMRI protocols identify convergent dysregulation across cognitive and emotional tasks within prefrontal networks [41]. Across anxiety disorders, meta-analytic work indicates altered medial PFC/rostral anterior cingulate cortex (ACC) engagement during emotional processing [42], and in generalized anxiety disorder specifically, resting-state analyses demonstrate reduced ventromedial prefrontal cortex (vmPFC)-insula functional connectivity, pointing to disrupted appraisal/interoceptive control loops [43].

#### Amygdala.

The amygdala, central to threat detection and emotional memory, shows heightened responsivity after sleep loss. A single night without sleep produces amplified amygdala responses to negative stimuli alongside reduced functional coupling with medial prefrontal regulatory regions—a prefrontal-amygdala “disconnect” [44]. Subsequent experimental work converges on this circuitry: partial sleep restriction (“sleep debt”) diminishes amygdala-anterior cingulate/medial PFC connectivity while increasing negative emotional reactivity [23].

#### Hippocampus.

The hippocampus underpins episodic and contextual memory and is a key node for sleep-dependent consolidation. Convergent evidence shows that slow-wave sleep (SWS) coordinates hippocampal sharp-wave ripples, thalamo-cortical spindles, and cortical slow oscillations to enable systems consolidation, i.e., gradual redistribution of memories from hippocampus to neocortex [45–47]. When sleep is curtailed or fragmented, hippocampal encoding and next-day recall degrade, and the hippocampus shows reduced support for new learning [46,48]. In clinical populations, structural hippocampal vulnerability intersects with sleep disruption: large-scale meta/mega-analyses report smaller hippocampal volumes in major depression [49] and PTSD [50]. Given that SWS is often reduced or fragmented across these disorders, impaired ripple-spindle-slow-oscillation coupling provides a plausible pathway from disturbed sleep to intrusive memories, impaired contextualization, and cognitive symptoms.

#### Hypothalamus (SCN, orexin/hypocretin).

The anterior hypothalamus houses the suprachiasmatic nucleus (SCN), the master circadian pacemaker that synchronizes sleep-wake timing via neural and endocrine outputs, including regulation of nocturnal melatonin release by the pineal gland. Circadian disruption—through SCN network alterations, clock-gene dysregulation, or misaligned zeitgebers—has been linked to mood and anxiety pathology [15,17,19]. Human imaging work further suggests altered SCN-centered connectivity in youth with depression and insomnia symptoms [18].

Adjacent hypothalamic orexin (hypocretin) neurons stabilize wakefulness and integrate arousal, stress, and reward signals; loss of orexin causes narcolepsy, highlighting its wake-promoting role. Pharmacologic blockade of orexin signaling (dual orexin receptor antagonists, DORAs) improves sleep onset and maintenance in insomnia, underscoring clinical leverage at this node [51,52]. Because orexin also interfaces with stress and reward circuits, dysregulation may contribute to hyperarousal in mood/anxiety disorders and to transdiagnostic sleep-psychopathology coupling [17,53].

### 3.2. Neurotransmitter systems

#### GABA.

GABA is the principal inhibitory transmitter governing sleep initiation and maintenance [54]. Convergent neurochemistry and pharmacology point to reduced GABAergic tone in insomnia and hyperarousal states, while many hypnotics (benzodiazepines, “Z-drugs”: Zolpidem, Zaleplon, and Eszopiclone) exert their effects as positive allosteric modulators at GABA_A receptors [55,56]. Magnetic resonance spectroscopy studies have reported lower cortical GABA in chronic insomnia, consistent with difficulty down-regulating arousal at night [55]. Clinically, augmentation of GABA_A signaling consolidates sleep but can shift non-rapid eye movement sleep (NREM) microarchitecture toward lighter stage N2, underscoring a trade-off between sedation and physiological sleep depth [55,56].

#### Serotonin (5-HT).

Serotonin modulates both mood and sleep architecture, especially the REM phase. Depressive states are linked to reduced serotonergic tone and characteristic REM changes (shortened REM latency, increased REM pressure), while selective serotonin reuptake inhibitor (SSRIs)/ serotonin-norepinephrine reuptake inhibitors (SNRIs) typically suppress REM and may fragment sleep early in treatment even as mood improves [57,58]. Beyond REM control, serotonergic pathways interface with circadian timing, helping entrain sleep-wake rhythms via raphe-SCN signaling [58].

#### Dopamine.

Dopamine promotes wakefulness, motivation, and salience. Mesocorticolimbic and nigrostriatal dopamine pathways increase arousal and shorten sleep, and dopaminergic activation by stimulants enhances alertness at the cost of longer sleep-onset latency and shorter total sleep time when taken later in the day [59]. In psychotic disorders, hyperdopaminergia contributes to positive symptoms and may destabilize sleep-wake regulation; antipsychotic D2-receptor blockade can secondarily improve sleep continuity while introducing dose-dependent sedation [59–62].

#### Orexin (Hypocretin).

Orexin neurons in the lateral hypothalamus stabilize wakefulness by tonically exciting arousal systems (monoaminergic and cholinergic). Loss of orexin signaling causes narcolepsy, illustrating its leading role in state stability; more subtle dysregulation has been implicated in hyperarousal phenotypes across mood/anxiety conditions [53]. Clinically, dual orexin receptor antagonists (DORAs) improve sleep onset and maintenance without GABAergic sedation, offering a mechanistically distinct option for insomnia and a window into the arousal-stress-reward nexus [52].

### 3.3. Circadian rhythms and sleep homeostasis

#### Suprachiasmatic nucleus and CLOCK genes.

The suprachiasmatic nucleus (SCN) serves as the brain’s master circadian pacemaker, regulating 24-hour rhythms via core clock genes such as CLOCK, ARNTL (BMAL1), PER1–3, CRY1–2, and NPAS2, which coordinate sleep-wake timing, hormonal secretion, and emotional regulation. Disruptions of these rhythms are consistently observed in mood and anxiety disorders, prompting early candidate-gene studies to examine associations between circadian genes (e.g., CLOCK, NPAS2, PER2/3, CRY2, RORA, NR1D1) and psychiatric illness [15–17,19,63]. However, the most recent and very large, hypothesis-free genome-wide association studies of major depressive disorder (MD)—a 2025 trans-ancestry meta-analysis of 688,808 cases and 4,364,225 controls—have identified 697 independent SNP associations at 635 loci but did not prioritize canonical clock genes among the 308 high-confidence genes associated with MD; instead, the hits were enriched for synaptic and neuronal signaling genes [64]. Similarly, a large GWAS of 41,917 individuals with bipolar disorder (BD) and 371,549 controls identified 64 risk loci enriched for synaptic and brain-expressed genes rather than circadian-clock genes [65]. These findings suggest that, at the level of common genetic variation detectable by current GWAS, core clock genes do not represent major direct risk loci for MD or BD. Rather, circadian disruption in psychiatric disorders may more often reflect systems-level dysregulation of SCN-mediated pathways, environmental and behavioral influences, epigenetic mechanisms, or rare variants not captured by GWAS—while circadian signaling may still meaningfully impact emotion-regulation circuits and remain a valid target for chronobiological interventions.

#### Genetic overlap and shared heritability.

Large-scale genome-wide association studies (GWAS) have revealed significant overlapping heritability between sleep traits and major mental illnesses [66,67]. For example, a recent meta-GWAS of insomnia (n > 1.3 million) identified hundreds of risk loci and found that polygenic risk for insomnia is strongly genetically correlated with major depressive disorder and related conditions [66]. Twin and family studies likewise show that much of the co-occurrence of disordered sleep and mood disorders is due to common genetic influences: the genetic correlation between insomnia (or poor sleep quality) and depression is often very high (r ≈ 0.6–0.8) in twin analyses [68], supporting the hypothesis that shared hereditary factors underlie both sleep disturbance and depression [69]. Notably, even normal variation in circadian rhythm and sleep duration shows genetic overlap with psychiatric risk: e.g., a predisposition toward “eveningness” (late chronotype) and longer habitual sleep are associated with higher genetic risk for schizophrenia and bipolar disorder, whereas depression, anxiety and post-traumatic stress disorder are linked to insomnia and shorter sleep [67]. Mendelian randomization analyses further bolster evidence of a causal interplay: genetic liability to insomnia increases the risk of developing depression (and vice versa) in bidirectional MR experiments [66,70]. Collectively, these findings indicate that overlapping biological pathways (such as those regulating sleep–wake cycles and affective brain circuits) may contribute to both sleep abnormalities and psychiatric disorders, explaining their frequent co-morbidity.

#### Circadian misalignment and psychiatric symptoms.

When internal rhythms drift from environmental or social time (shift work, irregular schedules, social jetlag, evening chronotype), risk for mood and anxiety symptoms may rise [19,71]. Meta-analytic and epidemiologic work links eveningness and social jetlag with higher depressive symptom burden and poorer mental health [19,71] while shift work is associated with greater odds of depression/anxiety [72]. Mechanistically, misalignment has been linked to altered endocrine profiles (e.g., cortisol awakening response), emotion-regulation difficulties, and reduced cognitive control [17,19].

On the other hand, sleep deprivation can produce rapid, short-lived antidepressant effects by acutely altering neural circuits and neurochemical systems implicated in depression. One night of wakefulness increases synaptic potentiation and glutamatergic signaling in prefrontal and limbic regions, enhancing plasticity in mood-regulatory networks [73,74]. It also boosts dopaminergic transmission in the mesocorticolimbic system, which can transiently improve reward processing and motivation [75,76]. Sleep loss increases adenosine activity and A1 receptor signaling, modulating monoamine pathways implicated in antidepressant response [77,78]. At the systems level, sleep deprivation rapidly adjusts circadian and clock-gene rhythms, helping realign mis-timed oscillators commonly observed in depression (Benedetti & Colombo, 2011; Wirz-Justice et al., 2013). Functional imaging studies also show reduced default mode network hyperconnectivity and improved prefrontal–amygdala regulation, mechanisms linked to lower negative affect [79,80]. These neurobiological shifts likely converge to produce the characteristic, but temporary, antidepressant response.

#### Social zeitgebers and behavioral rhythms.

External cues (zeitgebers) such as light, particularly morning daylight – the dominant entraining signal, along with meal timing, physical activity, and social contact, synchronize internal clocks; disruption of these routines may precipitate mood episodes [81]. Although CBT-I is the first-line treatment for sleep disorders, therapies that stabilize daily rhythms—most notably light therapy and Interpersonal and Social Rhythm Therapy (IPSRT)—improve outcomes in bipolar disorder [82] by regularizing sleep-wake timing and social schedules [83], with supportive data for delayed relapse and better mood stability in subsequent trials [84–86]. Together, these findings position rhythm assessment and zeitgeber hygiene as integral elements of routine psychiatric care.

### 3.4. Inflammatory and endocrine pathways

#### HPA axis hyperactivity and cortisol dysregulation.

Sleep, particularly deep slow-wave sleep (SWS), exerts an inhibitory influence on the hypothalamic-pituitary-adrenal (HPA) axis and cortisol secretion [87]. Conversely, acute and chronic sleep deprivation activate the HPA axis, increasing cortisol levels and promoting a state of central nervous system hyperarousal that undermines sleep continuity and promotes a vicious cycle [88–91]. This hyperactivity can be a contributing factor to clinical insomnia, while in other sleep disorders like obstructive sleep apnea (OSA), HPA axis hyperactivity may be a consequence of the disorder that then contributes to other pathologies [92].

The relationship between the HPA axis and psychiatric illness is more complex than simple hyperactivity. Research shows that while some individuals with PTSD exhibit elevated cortisol levels, others have abnormally low levels [93,94], pointing to broader HPA-axis dysregulation rather than a uniform up- or down-regulation [95,96]. This pathological imbalance may be influenced by genetic predispositions and early-life trauma [97,98]. Furthermore, research indicates that epigenetic modifications, such as DNA methylation, on HPA axis and inflammatory genes are consistently implicated in the pathophysiology of PTSD, providing a molecular basis for the lasting biological imprint of trauma [99,100].

#### Pro-inflammatory cytokines and sleep fragmentation.

Elevated C-reactive protein (CRP) and Interleukin-6 (IL-6) are consistently observed with sleep disturbance and with extreme sleep durations, and meta-analytic data in depression show increases in IL-6/ Tumor Necrosis Factor-Alpha (TNF-α) and CRP [101–104]. Inflammatory activation alters sleep architecture, often reducing slow-wave sleep and fragmenting continuity, as shown in human experimental endotoxin studies, clinical samples in which TNF-α blockade improves continuity, and population data linking higher IL-6 to shorter SWS [105–109]. Notably, a large meta-analysis found no consistent rise in CRP/IL-6 after acute sleep deprivation, implicating chronic disturbance as the driver of low-grade inflammation [104]. Conceptually, these links fit within the social signal transduction/sickness-behavior framework whereby inflammatory signaling shapes mood and behavior [110].

#### Bidirectional feedback between stress, inflammation, and sleep.

The HPA axis and pro-inflammatory signaling are not isolated systems but components of a shared, self-reinforcing feedback loop [90,111]. Sleep loss acutely activates inflammatory pathways and heightens stress reactivity, while persistent disturbance is associated with elevated CRP/IL-6. Even though acute deprivation alone does not reliably raise these markers, together they sustain a vicious cycle of hyperarousal and low-grade inflammation [104,109,112]. This perpetual feedback transforms an adaptive, short-term response into a chronic, pathological process that may contribute to disease progression and psychiatric relapse [90].

The mechanisms of this cycle are both systemic and cellular, involving key non-neuronal cells within the central nervous system. Chronic stress and sleep disruption bias microglia toward a pro-inflammatory state and alter astrocytic functions that gate sleep and metabolic clearance, contributing to fragmentation [113–115]. Chronic sleep fragmentation also impairs glymphatic transport, which is enhanced during deep sleep, thus promoting the accumulation of neurotoxic metabolites and further neuroinflammation [116–119].

### 3.5. Metabolic dysregulation

#### Insulin sensitivity and glucose regulation.

Metabolic disturbances emerge rapidly when sleep is curtailed or fragmented. A recent meta-analysis of controlled sleep-manipulation studies demonstrates that even short-term restriction significantly reduces whole-body insulin sensitivity and impairs glucose tolerance in healthy adults [120]. A large prospective cohort from the Multi-Ethnic Study of Atherosclerosis found that higher N3 proportion and longer N3 duration were associated with lower incident type 2 diabetes risk in older adults [121]. These alterations are relevant to psychiatric disorders: individuals with major depressive disorder show higher insulin resistance compared with healthy controls [122], and longitudinal data indicate a bidirectional relationship between depression and metabolic syndrome [123].

#### Appetite signaling and energy homeostasis.

Sleep loss alters endocrine mechanisms governing energy balance. Acute sleep restriction lowers leptin and increases ghrelin, shifting appetite toward greater caloric intake and preference for energy-dense foods [124]. Restricted sleep also elevates circulating endocannabinoids, enhancing reward-driven eating behaviors [125]. Appetite-related hormone dysregulation is clinically relevant: leptin and ghrelin abnormalities are documented in atypical depression and in antipsychotic-treated schizophrenia, where weight gain and altered appetite regulation are common [126,127]. Sleep loss may exacerbate these vulnerabilities by further altering leptin–ghrelin signaling.

#### Circadian control of metabolic processes.

Metabolic efficiency is strongly shaped by circadian phase. Forced-desynchrony and simulated shift-work experiments show that identical meals consumed during circadian misalignment result in higher postprandial glucose excursions and reduced insulin sensitivity compared with aligned conditions, even when sleep opportunity is held constant [128]. Core metabolic processes—including glucose tolerance, lipid oxidation, and resting energy expenditure—follow intrinsic circadian rhythms regulated by central and peripheral clocks; disruption of these rhythms impairs substrate utilization and reduces metabolic flexibility [129]. Circadian and metabolic disturbances co-occur in bipolar disorder: actigraphy data link sleep/circadian irregularities to metabolic syndrome components [130], and reviews highlight high rates of obesity, insulin resistance, and type 2 diabetes, conceptualized as “chrono-metabolic” or “metabolic jet lag” phenomena in bipolar illness [131,132].

#### Metabolic conditions that alter sleep physiology.

Metabolic dysfunction is highly prevalent in severe mental illness and is often amplified by second-generation antipsychotics, which substantially increase the risk of obesity and metabolic syndrome [133]; in schizophrenia, the presence of metabolic disorders is independently associated with poorer sleep quality and more severe sleep disturbance [134].

Metabolic dysfunction can, in turn, degrade sleep. Obesity increases upper-airway collapsibility and the likelihood of obstructive sleep apnea, producing recurrent arousals and intermittent hypoxia that further impair insulin sensitivity [135]. Diabetes-related hyperglycemia and nocturnal glucose variability correlate with reduced slow-wave sleep and increased nocturnal awakenings [136]. Interventions that improve metabolic health—including weight loss or treatment of sleep apnea—produce parallel improvements in sleep continuity and neurocognitive/mood outcomes [137].

## 4. Disorder-specific sections: Bidirectional sleep-psychopathology interactions

### 4.1. Major Depressive Disorder (MDD)

#### Prevalence and types of sleep disturbances.

Sleep disturbances are among the most prevalent and diagnostically significant features of major depressive disorder (MDD) [138]. According to DSM-5 criteria, individuals may present with either insomnia or hypersomnia as one of the A-criteria for a major depressive episode, underscoring the heterogeneity of sleep-related manifestations [30]. Epidemiological evidence indicates that sleep complaints occur in up to 90% of depressive episodes, with insomnia symptoms reported by about 85% in a large population-based sample and commonly manifesting as difficulty initiating or maintaining sleep or early-morning awakening [139]. Hypersomnia, while less common overall, appears more frequent in younger patients with depression [140] and is a hallmark of episodes with atypical features [141] and is also enriched in bipolar-spectrum depressions [142], suggesting distinct mechanisms and clinical trajectories.

#### Bidirectional effects of sleep and depression.

Prospective and meta-analytic studies indicate a robust, bidirectional relationship between insomnia and depression. Persistent insomnia is associated with approximately a 2- to 3-fold increased risk for incident depression and predicts a poorer course, with elevated relapse and recurrence risk when insomnia persists after mood remission [7–9,143]. Cognitive mechanisms help explain this coupling: rumination mediates the link between low mood and poor sleep quality [144], and nighttime cognitive intrusions (worry/intrusive thoughts) prospectively elevate depression risk via worsening insomnia [145,146]. A recent framework proposes a self-reinforcing triad: mind-wandering, sleep disruption, and negative affect, that maintains both insomnia and depressive symptoms [147].

#### Neurobiological mechanisms.

Foundational accounts emphasized monoaminergic dysregulation, particularly reduced serotonergic tone, as a shared substrate for depression and associated sleep abnormalities [20,148]. Characteristic rapid eye movement (REM) changes are among the most robust neurophysiological markers of depression: higher REM density consistently differentiates unmedicated patients with major depressive disorder (MDD) from healthy controls [149] and has been linked to greater depressive symptom severity in population-based samples [150]. Beyond monoamines, dysregulation of the hypothalamic-pituitary-adrenal (HPA) axis provides another mechanistic link between sleep and mood pathology. Large cohort studies show that individuals with MDD exhibit elevated cortisol awakening responses and altered diurnal secretion patterns [151], while experimental and clinical data suggest that corticotropin-releasing hormone and glucocorticoid excess contribute to disrupted sleep architecture [152,153].

Contemporary circuit-level work integrates these pathways with affect regulation. Functional MRI indicates that sleep disturbance and deprivation alter connectivity among prefrontal-limbic networks central to emotional control (including amygdala and insula) [44,154]. In first-episode, drug-naïve MDD with comorbid insomnia, resting-state analyses show broader prefrontal-limbic abnormalities with reduced left insula activity versus MDD without insomnia [155]. Poorer sleep quality is also linked to increased amygdala-subgenual anterior cingulate cortex (sgACC) connectivity in clinical samples, consistent with a hyperreactive affective circuit [156]. Emerging evidence also suggests slow-wave activity (SWA) may operate atypically in MDD: experimental disruption of slow-wave sleep selectively altered waking theta in depressed participants, consistent with an altered homeostatic process that could undermine mood regulation [157].

#### Interventions targeting sleep.

Targeting insomnia meaningfully improves both sleep and mood outcomes in MDD. CBT-I consistently enhances sleep and reduces depressive symptoms, and as an adjunct to usual care, it increases depression response (with remission gains varying by trial) [158–160]. Despite this, implementation remains limited; major guidelines recommend integrating sleep and chronobiology interventions, especially CBT-I, into routine care rather than assuming sleep will normalize with antidepressants alone [161,162]. Pharmacologically, SSRIs typically increase REM latency, suppress REM, and can initially worsen sleep continuity, warranting proactive management [57,163]. Low-dose trazodone is often used to mitigate SSRI-related insomnia, with benefits balanced against tolerability [164,165]. Emerging options include dual orexin receptor antagonism (e.g., seltorexant) for residual insomnia in antidepressant-treated MDD; phase 2 randomized, placebo-controlled multicenter trials report improvements in sleep and suggest possible adjunctive antidepressant effects, but sample sizes are modest and long-term data are lacking, so the evidence is promising but still preliminary [166,167].

### 4.2. Bipolar Disorder (BD)

#### Prevalence and types of sleep disturbances.

Sleep–circadian disturbance is a core feature of bipolar disorder, manifesting as reduced sleep need and insomnia during manic episodes and hypersomnia during bipolar depression [168,169]. Importantly, abnormalities such as delayed sleep phase, irregular sleep timing, and residual insomnia frequently persist into euthymia, reflecting a trait-like rhythm instability that contributes to relapse risk and functional burden [168,170,171].

#### Bidirectional effects of sleep and bipolar disorder.

Sleep and circadian disturbances in bipolar disorder are both markers and drivers of mood instability. Insomnia is a common prodromal feature and frequently precedes manic episodes, whereas hypersomnolence more often heralds depressive episodes [172]. Prospective syntheses and actigraphy studies show that instability in sleep—particularly night-to-night variability in timing and duration and irregular rest-activity rhythms—predicts relapse even during euthymia, while average sleep duration is less informative [173–176].

Sleep loss is a well-recognized trigger of mania [177], and therapeutic sleep deprivation for bipolar depression can rapidly improve mood but carries a risk of switching to mania or hypomania, with rates of 5–10%—comparable to those observed with antidepressant treatments [178].

#### Neurobiological mechanisms.

Circadian clock pathways show vulnerability in BD: polymorphisms in CLOCK and related genes (BMAL1/ARNTL, PER, CRY) align with altered sleep-wake timing [15,63], and epigenetic Brain and muscle Arnt-like protein-1 gene (ARNTL) methylation differences suggest rhythm misalignment at the molecular level [179]. Lithium engages these clocks, lengthening circadian period and increasing amplitude in cellular/animal models, which provides a mechanistic basis for rhythm stabilization [180,181]. These data align the pharmacology of a first-line mood stabilizer with core-clock modulation and help explain why interventions that target circadian organization can complement medication in BD.

#### Interventions targeting sleep.

Rhythm-focused care is central. IPSRT regularizes sleep/wake and other zeitgebers, delaying relapse and improving mood stability, although samples are modest and largely from specialized centers [83,86]. Chronotherapies, including bright light exposure (morning or midday), phase-advance protocols, and controlled sleep deprivation, can produce rapid antidepressant effects, particularly when paired with mood stabilizers and close monitoring for mood switches [82,182,183]. Adjunctive melatonin or ramelteon has shown modest and mixed benefits, with small trials suggesting possible improvements in sleep and circadian alignment and potential relapse prevention, though evidence remains limited [184,185]. In acute manic or depressive episodes, sedating second-generation antipsychotics such as olanzapine, asenapine, and risperidone tend to increase total sleep time and improve sleep continuity in addition to stabilizing mood, based primarily on secondary sleep outcomes from controlled trials and small polysomnographic studies rather than dedicated sleep RCTs [186,187].

### 4.3. Anxiety disorders

#### Prevalence and types of sleep disturbances.

Clinically significant sleep disturbance is the norm across anxiety disorders. In generalized anxiety disorder (GAD), insomnia is reported by up to ~90% of patients, driven by persistent worry and cognitive hyperarousal [188]. Panic disorder is marked by insomnia, nocturnal panic attacks, and fragmented sleep; meta-analysis shows longer sleep latency, poorer efficiency, and shorter total sleep time, with over half of patients reporting at least one nocturnal panic episode [189], findings echoed by early polysomnography documenting reduced efficiency and frequent awakenings [190].

#### Bidirectional effects of sleep and anxiety disorders.

Sleep and anxiety amplify each other. Prospective data suggest sleep problems more strongly predict later anxiety than vice versa [191], and chronic insomnia forecasts elevated anxiety, potentially via inflammatory pathways [192]. Polysomnography in large samples links higher state/trait anxiety with lighter sleep and reduced SWS [193]. Longitudinal work also shows insomnia and anxiety co-predict over time [194].

#### Neurobiological mechanisms.

***REM-related mechanisms (extinction and safety learning)*:** REM sleep supports emotional-memory updating, particularly fear extinction and safety learning. Causal rodent evidence shows that infralimbic medial prefrontal cortex (mPFC) activity during REM is required to consolidate extinction [195]. In humans, greater REM duration relates to better safety-signal learning in trauma-exposed veterans, while reduced REM predicts stronger fear-potentiated startle in PTSD [196,197]. Consequently, REM fragmentation, frequent in GAD/PTSD, likely weakens extinction consolidation and may blunt gains from exposure-based therapies [198].

***Endocrine and noradrenergic mechanisms*:** HPA-axis dysregulation and heightened locus coeruleus–norepinephrine (LC–NE) output contribute to sleep disruption across anxiety presentations, with elevated nighttime sympathetic activity impairing both REM continuity and restorative NREM sleep [199–204].

#### Interventions targeting sleep.

CBT-I improves insomnia and anxiety outcomes in comorbid GAD, with sleep gains often preceding or predicting reductions in worry, panic, and avoidance [205,206]. For panic disorder, CBT tailored to nocturnal panic reduces night awakenings and panic severity [207]. Hypnotic augmentation strategies, such as eszopiclone combined with SSRIs [208], also demonstrate potential benefits when used selectively. Together, these findings underscore that treating sleep disturbance is a clinically meaningful pathway for improving anxiety symptoms.

### 4.4. Post-Traumatic Stress Disorder (PTSD)

#### Prevalence and types of sleep disturbances.

In post-traumatic stress disorder, disturbed sleep is diagnostically central—nightmares, insomnia, and hyperarousal are core DSM-5 criteria [30]. Objective monitoring consistently demonstrates abnormalities. Actigraphy indicates lower sleep efficiency, more fragmentation, and longer time in bed compared with controls [209]. Polysomnography reveals reduced total sleep time, diminished slow-wave sleep, and elevated REM density and fragmentation [35]. Nightmares and REM-related dysregulation are especially prominent and are among the most persistent symptoms across the illness course.

#### Bidirectional relationship between PTSD and sleep.

A robust longitudinal literature supports a bidirectional relationship between sleep and PTSD. In military and Veteran cohorts, pre-deployment sleep disturbance—particularly insomnia and nightmares—predicts post-deployment PTSD symptoms, indicating that disturbed sleep functions as a vulnerability factor rather than a mere consequence [210,211]. Large prospective military and Veteran cohorts, in which pre-deployment insomnia and nightmares predict post-deployment PTSD and PTSD predicts later onset of insomnia and obstructive sleep apnea [212,213], provide the most robust temporal evidence because they use repeated measures and adjust for baseline symptoms and deployment exposures. Among treatment-seeking veterans, insomnia and nightmares are longitudinally associated with higher PTSD severity and tend to persist even when daytime PTSD symptoms partially improve, suggesting that sleep disturbance is both a maintaining factor and a marker of incomplete recovery [214,215]. Beyond deployment samples, adverse childhood experiences and documented maltreatment show dose–response, long-term associations with insomnia and other sleep disturbances from adolescence into adulthood, supporting enduring effects of early trauma on sleep-regulatory systems [216,217].

#### Neurobiological mechanisms.

PTSD is characterized by intertwined alterations in fear circuitry, REM regulation, the HPA axis, and noradrenergic signaling. Nightlong recordings show more awakenings and higher heart rate, with awakenings positively related to adrenocorticotropic hormone (ACTH) and both ACTH/cortisol inversely related to slow-wave sleep [199]; this pattern aligns with corticotropin-releasing factor (CRF) hypersecretion and relatively low cortisol due to enhanced negative feedback sensitivity [200,201]. Evidence of elevated nocturnal central noradrenergic activity has been demonstrated in combat-related PTSD, where nocturnal 3-methoxy-4-hydroxyphenylglycol (MHPG) levels fail to show the normal nighttime decline and correlate with reduced sleep time [202]. Locus coeruleus (LC) output further suppresses REM-generating circuits in animal models: down-regulating NE synthesis increases REM, while local NE infusion into the pedunculopontine tegmentum prevents it [203]. Consistent with these mechanistic findings, neuromelanin-sensitive MRI reveals elevated LC signal in military PTSD, most pronounced in the caudal LC and correlating with hyperarousal severity [204].

#### Interventions targeting sleep.

CBT-I effectively improves insomnia and sleep efficiency in PTSD, often with parallel reductions in PTSD symptom severity [218]. For nightmares and REM-related hyperarousal, prazosin shows mixed efficacy—positive RCTs demonstrating sleep and nightmare improvements [219,220] versus a large negative trial [221]—but remains a targeted option for select patients. Complementary approaches such as brief behavioral treatment for insomnia (BBTI) in trauma-exposed populations [222], Continuous Positive Airway Pressure (CPAP) for comorbid sleep apnea [223], and hypnotic augmentation strategies like eszopiclone plus SSRI [208] further underscore the clinical value of addressing sleep directly as part of PTSD management.

### 4.5. Schizophrenia and psychotic disorders

#### Prevalence and types of sleep disturbances.

Sleep disturbance is the rule rather than the exception across the psychosis spectrum: ~ 63% of people with schizophrenia report poor sleep quality, conferring a ~ 4 × higher risk versus controls [224]. Across clinical high risk, early psychosis, and chronic stages, pooled prevalence is ~ 50%, with consistently poorer subjective sleep than in non-psychiatric samples [225]. Typical complaints include prolonged sleep latency, frequent awakenings, shortened total sleep time, and non-restorative sleep; polysomnography (PSG) corroborates longer sleep latency, more wake after sleep onset, and reduced efficiency, with greater fragmentation (and more severe spindle deficits) in chronic illness [225,226].

#### Bidirectional effects of sleep and psychotic symptoms.

Poor sleep quality degrades cognition, emotion regulation, and reality monitoring, amplifying persecutory ideation and other positive symptoms [227]. Within-person data show that worse efficiency/longer latency predicts next-day increases in hallucinations and disorganization, although these findings come from small, intensively monitored samples [228,229]. Conversely, active psychotic symptoms and hyperarousal disrupt initiation and continuity of sleep and may perturb melatonin rhythms, creating a self-reinforcing loop [228]. In high-risk youth, actigraphic sleep disturbances—decreased efficiency, increased wake after sleep onset (WASO) and awakenings, and nocturnal movements—predicted escalation of positive symptoms over 12 months [31]; in schizophrenia-spectrum disorders, a large eight-year cohort showed that baseline insomnia (especially when combined with nightmares) independently predicted suicide risk over eight years [230].

#### Neurobiological mechanisms: spindles and circuits.

Reductions in stage-2 sleep spindles are a robust electrophysiological signature, with large effects and associations with longer illness duration and cognitive deficits [226]. High-density electroencephalography (EEG) demonstrates marked decreases in spindle number, amplitude, and duration [231], and lower spindle density/coherence predicts impaired overnight memory and greater positive-symptom burden [232]. Converging neuroimaging shows thalamocortical dysconnectivity—reduced thalamo-prefrontal coupling alongside relative hyperconnectivity with sensorimotor/auditory networks—implying relay dysfunction that may disrupt both cognitive processing and sleep rhythms [233–235]. Dysconnectivity extends to basal ganglia circuits [236], and animal work indicates selective loss of thalamic glutamatergic input to auditory cortex driven by elevated D2 signaling, offering a circuit-level mechanism relevant to sensory gating and arousal regulation [237]. In sum, converging evidence links disrupted sleep spindles to broader thalamocortical and basal ganglia dysconnectivity, offering a unifying explanation for how a single circuit-level disturbance can underlie both impaired sleep physiology and the cognitive and clinical symptoms of psychotic illness.

#### Interventions targeting sleep.

Second-generation antipsychotics (SGAs) exert variable effects on sleep. SGAs such as olanzapine and quetiapine can improve total sleep time and continuity—effects plausibly linked to histamine-1 (H₁) and 5-HT₂ receptor antagonism—while clozapine appears to stabilize non-REM sleep without altering REM [238] Clinically, olanzapine and risperidone are associated with fewer sleep complaints than quetiapine or aripiprazole, yet residual insomnia, short sleep, and daytime sedation remain common, underscoring the limits of medication alone [239].

Adjunctive CBT-I in psychosis reliably reduces insomnia severity and improves sleep quality, with modest but meaningful reductions in psychotic symptoms across short- and long-term follow-up [240]. Treatment response is heterogeneous—higher baseline symptom burden and mood disturbance predict weaker functional gains—underscoring the value of personalized CBT-I delivery [241].

### 4.6. ADHD and neurodevelopmental disorders

#### Prevalence and types of sleep disturbances.

Sleep problems are highly prevalent in ADHD and other neurodevelopmental conditions, including ASD and intellectual disabilities. In ADHD, approximately 25–55% report clinically significant sleep disturbance even without other psychiatric comorbidity, most often delayed sleep onset, short sleep duration, bedtime resistance, and increased nocturnal motor activity [242]. Polysomnography in untreated adults with ADHD also shows alterations in sleep architecture—e.g., longer slow-wave sleep—though objective findings vary across age groups and studies [243,244].

In ASD, sleep difficulties are reported in up to ~80% of individuals, with frequent problems initiating and maintaining sleep, sleep-disordered breathing, and restless leg syndrome [245,246]. Pediatric sleep-lab cohorts confirm elevated rates of insomnia, circadian rhythm disorders, and nonspecific restlessness relative to non-ASD peers, and point to increased sleep-disordered breathing and restless legs in autistic populations, possibly related to sensory sensitivities and melatonin dysregulation [245,247].

#### Bidirectional effects of sleep and neurodevelopmental symptoms.

Delayed sleep-wake timing is common and clinically relevant. In adults with ADHD, indications of delayed sleep phase syndrome (DSPS) are markedly overrepresented (26% vs 2% in controls), alongside shorter and later sleep that correlate with hyperactivity and mood seasonality [248]. In adolescents, however, moderate/high DSPS risk is equally frequent in ADHD and controls—about one-third of both groups—suggesting that while DSPS may serve as a diagnostic marker in adults, in younger populations it reflects a broader transdiagnostic vulnerability [249]. Longitudinal data show reciprocal associations: insufficient or delayed sleep forecasts next-day hyperactivity/impulsivity and broader behavioral difficulties, and daytime symptoms predict that night’s sleep disruption [250,251]. Conceptually, a delayed circadian phase may even masquerade as “late-onset ADHD” in teens, underscoring shared mechanisms between circadian misalignment and attentional dysregulation [252].

In ASD, trajectories are nuanced. In high-functioning children, reductions in sleep problems over one year accompany improved social functioning, whereas baseline sleep disturbance predicts later anxiety [253]. In a larger ASD cohort, persistent sleep problems were linked to later ADHD symptoms (in younger children) and somatic complaints (in older children), with sensory over-responsivity prospectively predicting future sleep difficulties [254]. Population-based data further suggest that early sleep problems do not independently drive increases in autistic traits once baseline symptoms are considered; instead, higher autistic traits and ASD diagnoses predict more persistent or worsening sleep problems, implying that sleep disturbance may be integral to the ASD phenotype rather than a primary upstream cause [255].

#### Neurobiological mechanisms.

Multiple systems implicated in ADHD and ASD overlap with sleep regulation. In ASD, clinical and animal studies point to dysregulation of key neuromodulatory circuits that govern arousal and sleep, including the noradrenergic locus coeruleus, serotonergic dorsal raphe, dopaminergic ventral tegmental area, and histaminergic tuberomammillary nucleus [37,38]. These disruptions have downstream effects on NREM continuity, spindle dynamics, REM expression, and daytime behavior. Importantly, experimental studies show that improving sleep can reduce stereotypies and enhance social functioning, highlighting mechanistic links between sleep circuits and core symptoms [256].

Gene-to-circuit evidence implicates GABAergic, histaminergic, dopaminergic, serotonergic, and orexinergic pathways—via mutations in MECP2, VGAT, SLC6A1, HRH1–3, SLC6A3 genes, and others—in hyperarousal, prolonged awakenings, REM disruption, and circadian instability [38]. Melatonin pathway disruption is especially salient: acetylserotonin O-methyltransferase (ASMT) promoter/splicing variants are associated with markedly reduced ASMT expression/activity and low melatonin levels in ASD, supporting melatonin deficiency as a risk factor with implications for sleep timing and behavior [257]. Broader circadian/autonomic alterations (e.g., CLOCK-related changes, sympathetic hyperarousal, HPA-axis dysregulation) further connect sleep pathology with autism-specific symptom clusters [258].

#### Interventions targeting sleep.

Behavioral approaches—sleep hygiene, structured routines, and parent-mediated programs—show benefit and should be first line, particularly in younger children [259]. In ADHD, stimulants can either improve or impair sleep: by reducing evening restlessness, they may facilitate bedtime, but dosing close to bedtime delays sleep onset and shortens total sleep; adjusting timing/formulation or considering non-stimulants (e.g., guanfacine, atomoxetine) can mitigate sleep costs [242,260].

Melatonin is an evidence-supported option for delayed sleep phase and insomnia in both ADHD and ASD, with favorable long-term safety. Trials document improvements in sleep onset latency, total sleep time, and parent-rated outcomes; in ADHD, benefits were observed when melatonin was combined with structured sleep hygiene [261], and long-term follow-up confirms sustained efficacy and safety [262]. In ASD, meta-analytic and trial data demonstrate that both immediate- and prolonged-release melatonin formulations shorten sleep latency, reduce night awakenings, extend total sleep time, and improve associated daytime behaviors [263–265].

### 4.7. Substance Use Disorders (SUDs)

#### Prevalence and types of sleep disorders.

Clinically significant sleep problems are the rule rather than the exception across substance use disorders (SUDs). In alcohol use disorder (AUD), PSG and meta-analytic evidence show longer sleep onset latency, lower sleep efficiency, reduced slow-wave sleep (SWS), and heightened REM pressure/REM density—abnormalities that can persist into abstinence [266,267]. Cocaine- and alcohol-dependent cohorts similarly exhibit marked SWS loss and increased REM, with these age-related changes occurring earlier than in controls [268]. Among stimulant users, poor sleep quality and curtailed sleep duration are common, and in cocaine dependence, these disturbances have been linked to stronger craving and earlier relapse [269,270].

Population data from Chinese illicit drug users underscore the breadth of the problem: 68.5% screened positive for sleep disturbance (Pittsburgh Sleep Quality Index (PSQI)>5), and 43.9% reported poor sleep quality (PSQI>8), with higher PSQI scores in heroin users and a dose–response association with longer use duration [271]. In methamphetamine withdrawal, sleep is especially impaired early (97.8% poor sleepers in week 1) but improves substantially by week 4 of abstinence (52.2%), largely independent of changes in mood symptoms [272]. In opioid use disorder (OUD), > 75% report multidimensional sleep deficiency (satisfaction, timing, efficiency, duration), reflecting a problem that is both prevalent and clinically consequential [273].

#### Substance-specific effects on sleep.

Many of the substances involved in substance use disorders have characteristic and often bidirectional effects on sleep. Alcohol is acutely sedating at sleep onset but RCTs, systematic reviews, and prospective AUD cohorts consistently show that it disrupts sleep architecture—suppressing REM and deep sleep early in the night, increasing light sleep and fragmentation, and producing REM rebound and persistent insomnia with long-term use, with chronic alcohol use disorder showing enduring macro- and microarchitectural abnormalities linked to mood and cortical atrophy [274–276]. Cannabinoids modulate sleep in a dose- and formulation-dependent manner: Δ9-THC can shorten sleep latency and acutely increase slow-wave sleep but, with chronic or heavy use and during withdrawal, is linked to poorer subjective sleep quality, reduced total sleep time, and insomnia complaints, with polysomnographic data showing heterogeneous effects on staging [277–279]. Opioids, including in the context of opioid use disorder and chronic opioid therapy, fragment sleep, reduce slow-wave and REM sleep, and increase the risk of sleep-disordered breathing and daytime sleepiness; sleep deficiency in OUD is now recognized as both a consequence of opioid exposure and a predictor of craving and relapse [280,281]. Stimulants such as cocaine and amphetamines shorten sleep time, prolong sleep onset latency, and suppress REM, whereas early abstinence is characterized by hypersomnia, REM rebound, and persistent insomnia, with chronic use producing long-lasting disruption of sleep architecture and circadian timing [282–284]. Finally, nicotine from combustible and electronic cigarettes is associated with longer sleep latency, shorter sleep duration, and poorer subjective sleep quality, with systematic reviews and observational data indicating higher rates of insomnia and sleep disturbance in both traditional smokers and e-cigarette users [285–287]. Together, these substance-specific alterations in sleep architecture and continuity both contribute to and are maintained by the underlying addiction, reinforcing the bidirectional relationship between sleep disruption and substance use disorders.

#### Bidirectional effects of sleep and substance use disorders.

Sleep disturbance is not just epiphenomenal—it predicts worse substance outcomes. Poor baseline sleep in stimulant users tracks with heavier recent use [288] and, across substance use disorders more broadly, poorer nightly sleep quality predicts stronger next-day craving [289]. In AUD, persistent insomnia after detoxification doubles relapse risk over subsequent months [290]. In OUD, higher insomnia severity at treatment intake predicts return to use and non-fatal overdose; symptom improvement during treatment tracks with better outcomes, and persistent insomnia over the first six months portends relapse risk [32]. More broadly, lifetime insomnia and hypersomnia map onto distinct, higher-risk substance patterns and predict increased cocaine use/relapse [291]. These data support a reciprocal cycle in which sleep disruption sustains drug seeking/use, and ongoing use further degrades sleep [267].

#### Neurobiological mechanisms.

Converging human and preclinical work points to overlapping circuitry for sleep, reward, stress, and pain. In AUD, sleep pathology aligns with the addiction cycle: during intoxication, faster sleep onset but poorer sleep; during withdrawal, SWS loss and only partial REM recovery; during protracted abstinence, persistent insomnia, reduced delta power, and heightened REM—changes linked to adaptations in GABA/glutamate, dopamine, stress systems (corticotropin-releasing factor, norepinephrine, orexin), and circadian regulation [36,267]. In opioid states, sleep deficiency amplifies stress signaling and hyperalgesia, while opioids directly disrupt ventilatory control during sleep, increasing central and obstructive apnea, and thereby fragmenting sleep [273]. The orexin (hypocretin) system—integrating arousal, stress, and reward—is increasingly implicated across SUDs; its dysregulation contributes to sleep fragmentation and craving, and dual orexin receptor antagonists (DORAs) are under active investigation [273,292].

#### Interventions targeting sleep.

Routine assessment of sleep (including insomnia, circadian timing, and sleep-disordered breathing) should be part of SUD care, with early intervention to reduce relapse risk [32,290]. Cognitive Behavioral Therapy for Insomnia (CBT-I) is effective in SUD samples and feasible to implement in outpatient addiction programs, though access and adherence can be barriers [293,294].

Pharmacologic options may be considered case-by-case: gabapentin can improve sleep and drinking outcomes in subsets of AUD [295,296], and acamprosate may confer modest sleep benefits alongside anti-relapse efficacy [297]. Sedative-hypnotics warrant caution in AUD/SUD given misuse risk and potential for poor-quality sleep [267]. Novel targets include orexin antagonists for sleep/craving modulation and cannabidiol (CBD) as a non-intoxicating candidate for AUD-related sleep disturbance with an encouraging safety profile, though controlled trials remain limited [292,298].

Finally, timing matters: acute stimulant withdrawal may show meaningful sleep recovery within weeks, while alcohol- and opioid-related sleep pathology can endure for months, reinforcing the need for staged, disorder-specific sleep strategies [36,272,273].

### 4.8. Other psychiatric disorders

#### Prevalence and types of sleep disorders.

This section outlines sleep disturbances in less commonly studied psychiatric disorders. In obsessive-compulsive disorder (OCD), sleep complaints are common, but evidence suggests they are largely driven by comorbid depression and trait anxiety [299]. Patients with OCD without depression show sleep patterns similar to controls, whereas higher levels of depression and anxiety independently predict poor sleep quality [299,300].

In eating disorders (EDs), anorexia nervosa (AN) shows shortened total sleep time and greater wake after sleep onset, increased stage 1 sleep, and reduced REM; notably, weight restoration alone may not normalize these abnormalities [301]. Bulimia nervosa (BN) commonly features irregular sleep-wake schedules and circadian instability [302,303].

In borderline personality disorder (BPD), meta-analytic evidence indicates longer sleep-onset latency, lower sleep efficiency, and altered REM parameters, alongside elevated self-reported sleep problems [304]. In younger populations, subjective complaints of poor sleep and insomnia symptoms are pronounced, though actigraphy suggests relatively longer and more efficient sleep compared to clinical controls, highlighting a subjective-objective sleep discrepancy [305]. Somatic symptom disorder is characterized by frequent insomnia—reported in approximately 20–48% of patients—alongside other sleep complaints such as non-restorative sleep and difficulty maintaining sleep [306].

#### Bidirectional effects of sleep and psychopathology.

Across these disorders, sleep disruption and core symptoms reinforce one another. In OCD, poorer or delayed sleep prospectively relates to more intrusive thoughts and worse emotional regulation, while evening rituals and rumination further postpone sleep [299,307]. In EDs, insufficient or irregular sleep contributes to appetite dysregulation and impulsive eating, and disordered eating patterns destabilize circadian timing [303,308]. In BPD, sleep disturbances are consistently associated with symptoms and functional impairment [309], and early childhood sleep problems have been linked to later BPD [310]. In somatic symptom disorders, sleep disturbance is closely linked with greater symptom severity and functional impairment, and heightened bodily attention and somatic complaints such as pain or gastrointestinal discomfort further interfere with sleep, reinforcing a bidirectional cycle [306].

#### Neurobiological mechanisms.

Shared neurobiological mechanisms span hyperarousal, circadian misalignment, and interoceptive amplification: in OCD, convergent imaging/review data implicate cortico-striato-thalamo-cortical (CSTC) circuit abnormalities consistent with arousal dysregulation [311–313]. In eating disorders, hypothalamic/orexin pathways link arousal with energy balance; higher plasma orexin-A associates with poorer sleep quality in anorexia nervosa and in obesity, with broader sleep/circadian disruption summarized by recent reviews [303,314]. In borderline personality disorder, mechanistic work indicates a circadian phenotype alongside fronto-limbic (amygdala-prefrontal) alterations compatible with unstable sleep-wake regulation [315,316]. In somatic symptom-spectrum presentations, meta-analytic neuroimaging highlights heightened engagement of interoceptive/salience networks (insula/anterior cingulate), offering a neural basis for non-restorative, fragmented sleep [317].

#### Interventions targeting sleep.

In OCD, screen for delayed sleep timing because it is associated with poorer exposure and response prevention therapy (ERP) response [318]. If insomnia emerges, manage per insomnia guidance: consider CBT-I and optimize antidepressant choice/timing—avoid activating agents near bedtime; use sedating augmentation at low dose timed before bed when appropriate [57]. In eating disorders, assess sleep and circadian timing; patients show eveningness and impaired sleep, and ED treatment can improve sleep quality. Circadian-supportive measures (regular routines, light and meal timing) are theoretically justified, but direct ED trials of CBT-I or specific circadian protocols are limited [319]. In BPD, morning bright-light therapy shows preliminary circadian and mood benefits in small crossover/open-label studies, and no randomized controlled trials (RCT) of bright light therapy (BLT) or other sleep/chronotherapy exist to date [315]; guided digital CBT-I as an adjunct to dialectical behavior therapy (DBT) is under evaluation [320]. In somatic symptom disorders, prioritize CBT-I alongside graded activity and interoceptive psychoeducation, minimizing polypharmacy [306,321].

## 5. Clinical implications

### 5.1. Routine assessment and under-treatment

Sleep disturbance is an active driver of psychopathology, not merely epiphenomenal. Routine screening for insomnia, hypersomnia, circadian misalignment, sleep apnea risk, nightmares, and poor sleep quality should be embedded in every psychiatric assessment. Yet surveys and guideline reviews indicate under-recognition and under-treatment across services, with insomnia rarely treated with first-line behavioral care and often defaulting to sedatives [322]. Brief validated tools (e.g., (ISI), PSQI, STOP-BANG) and a 2–3-minute sleep history (sleep timing/regularity, latency, awakenings, daytime impairment, caffeine/alcohol, shift work, screens) can normalize detection and triage.

When a sleep disorder is suspected, add targeted steps: use STOP-BANG if there is snoring, obesity, or sedative use; collect a one-week sleep diary (or actigraphy if a circadian phase disorder is likely or diaries are unreliable); screen trauma-exposed patients for nightmares; and consider sleep-medicine referral or polysomnography when signs suggest OSA, parasomnia, or central hypersomnolence.

### 5.2. Treating sleep improves psychiatric outcomes

A growing body of evidence demonstrates that targeting insomnia produces benefits that extend beyond sleep itself. Adjunctive CBT-I [159,240,323–325,326], IPSRT [83,84,86,327], bright-light therapy [328], and chronotherapeutic interventions, including carefully timed light exposure, phase-advance protocols, and controlled sleep deprivation with recovery sleep [328–330,331], improve both sleep and broader psychiatric outcomes when added to standard treatment. Mindfulness-based interventions also show supportive evidence for enhancing sleep and contributing to symptom reduction in several psychiatric disorders [332–334].

### 5.3. Digital sleep tracking and objective measures

Out-of-lab objective monitoring supports personalized care and longitudinal tracking. Actigraphy is validated for estimating sleep duration, fragmentation, and circadian phase in clinical research and is practical in psychiatric populations [335,336]. Consumer wearables show improving agreement for total sleep time and timing but are less reliable for staging; clinicians should interpret device outputs cautiously and prioritize trends over absolutes [337]. Digital CBT-I (dCBT-I) scales access and improves sleep and mental health outcomes at the population level, facilitating stepped-care models [338].

### 5.4. Challenges with pharmacologic sleep aids in psychiatric populations

Medication for insomnia in psychiatric care is fraught with trade-offs: diagnostic heterogeneity, polypharmacy, substance-use risk, and vulnerability to next-day cognitive and motor impairment narrow therapeutic window. When a drug is indicated, it should be adjunctive to CBT-I, targeted to the primary complaint (sleep-onset versus maintenance; circadian delay/misalignment), time-limited, and paired with a deprescribing plan [161,339].

#### Agent selection.

For sleep-onset or circadian problems, prefer ramelteon or melatonin [340,341]. For sleep-maintenance or early-morning awakenings, low-dose doxepin (3–6 mg) is appropriate [342]. For mixed onset-and-maintenance presentations, consider a dual orexin receptor antagonist (DORA) [343,344]. These options still carry residual risks—chiefly next-day somnolence—and require monitoring for interactions with psychotropics [161,339].

Notably, the American Academy of Sleep Medicine (AASM) guidelines emphasize the limited strength of evidence supporting pharmacologic treatments for chronic insomnia. Although agents such as low-dose doxepin and the orexin receptor antagonist suvorexant are commonly used, the guidelines indicate that none of the currently available medications carry high-quality evidence or strong recommendations for routine management [340]. This further underscores the importance of prioritizing behavioral, circadian, and systems-based interventions that more directly address underlying mechanisms.

#### Older adults.

Avoid benzodiazepines and Z-drugs for insomnia; if medication is needed after CBT-I, consider low-dose doxepin (≤6 mg), ramelteon, or a DORA, and keep use short-term [161,339,345].

#### Higher-risk and off-label options.

Reserve benzodiazepines and Z-hypnotics for brief, time-limited use due to dependence, falls, and cognitive/motor impairment risks—especially in late life [161,345,346]. Do not use antipsychotics (e.g., quetiapine) solely for insomnia because of metabolic and neurologic harms [347]. Condition-specific choices demand nuance: prazosin reduces PTSD-related nightmares in some trials but not others [221,348]. Gabapentin may help when insomnia co-occurs with pain or alcohol use disorder but is not first-line and requires misuse-risk assessment [295].

#### Safety checks.

Evaluate and treat obstructive sleep apnea before initiating any sedative, and minimize sedatives in untreated apnea or respiratory compromise [349]. Review drug-drug interactions systematically: avoid ramelteon with strong (CYP1A2) inhibitors such as fluvoxamine [350]; use dose limits or avoid DORAs with CYP3A inhibitors/inducers [351]; and note that doxepin exposure increases with CYP2D6/2C19 inhibitors [352].

#### Deprescribing.

Taper the hypnotic, maintain CBT-I gains, and reassess monthly, consistent with guideline recommendations to minimize long-term hypnotic use [339,353].

### 5.5. Equity, lifespan, and sex differences

Sleep care should account for structural barriers—shift work, housing instability, and limited access to CBT-I/dCBT-I—by offering low-burden digital options, flexible scheduling, brief navigator support, and integration within primary care, addiction care, and community mental health [338,354]. Adolescents commonly show circadian delay [355]. The peripartum period is vulnerable to insomnia symptoms and circadian disruption [356,357]. Older adults face greater fall and cognitive risk with sedatives, so non-pharmacologic strategies and the lowest effective doses are preferred [345]. These differences should inform screening, timing of interventions, and medication choice.

## 6. Discussion

To integrate the evidence reviewed above, we provide two summary tables that consolidate the main transdiagnostic and disorder-specific sleep disturbance patterns. Table 1 summarizes common transdiagnostic sleep phenotypes, including their operational definitions, typical diagnostic contexts, and brief mechanistic and functional notes. Table 2 outlines disorder-linked patterns across MDD, BD, anxiety disorders, psychosis, SUD, ADHD/ASD, OCD, and eating disorders, including PSG/actigraphy signatures and circadian profiles.

**Table 1:** Common types of sleep disturbance (transdiagnostic patterns).

Phenotype	Operational definition	High-prevalence diagnoses	Mechanistic notes	Functional impact
Insomnia (initiation/ maintenance/ early-morning awakening)	≥3 nights/ week for ≥3 months with daytime impairment [30]	Very common in MDD [139], BD [168], GAD [188], PTSD [358], and frequent in psychosis [3–5] and SUD [36]	Hyperarousal—HPA/ immune and cognitive arousal [55,104]	↑ relapse risk, ↓ QoL/ function [7,9]
Hypersomnia/ excessive sleepiness	Prolonged sleep/ sleepiness despite adequate opportunity [30]	Prominent in atypical depression [141] and bipolar depression [142,168,169]; present in MDD subsets [140]	Homeostatic/ circadian and behavioral contributors [20]	Functional impairment and course heterogeneity [30]
Circadian delay/ misalignment	Late phase, irregular timing, social jetlag [30]	Phase delay/instability in BD and remitted schizophrenia [225,226]; delay/irregularity also in BD and ADHD [168,242,249]	SCN/clock and social-zeitgeber disruption [15,17,19]	Mood lability, cognitive inefficiency [71]
Poor sleep quality/ non-restorative sleep	Elevated PSQI; unrefreshing sleep [359]	Typical across depression, PTSD, psychosis, SUD [144,266,269–271]	Links to inflammation/ autonomic dysregulation [104]	Worse health and functioning [3–5]
REM/ NREM architecture abnormalities	PSG: ↓ REM latency, ↑ REM density; ↓ SWS; ↓ sleep efficiency (SE)	Affective disorders and PTSD show REM/SWS changes [35,149,158,198]; schizophrenia shows ↓ efficiency and stage abnormalities [225,226]; PTSD shows REM fragmentation/nightmares [35]; ASD/ADHD subsets show spindle/ dim light melatonin onset (DLMO) or SWS differences [38,243,245]	REM pressure & NREM microstructure alterations	Affective reactivity, memory deficits [47]

**Table 2:** Condition-linked patterns.

Disorder/group	Salient sleep features	PSG/ Actigraphy signatures	Circadian profile	Clinical notes
Major Depressive Disorder	Insomnia and/or hypersomnia are criterion-level; insomnia highly prevalent in episodes [30,139]	↓REM latency, ↑ REM density, ↓ SWS [149]	Eveningness/social jetlag linked to higher depressive burden [71]	CBT-I improves sleep and reduces depressive symptoms; adjunctive use increases response [158–160]
Bipolar Disorder	↓Need for sleep in mania/ hypomania; hypersomnia in bipolar depression [30,142]	Stage-dependent EEG/PSG deviations across mood states [168,169]	Actigraphy shows phase delay/instability is common [168,169,176]	Rhythm stabilization (e.g., IPSRT) improves outcomes [83,84]
Anxiety disorders	High rates of insomnia/ poor sleep quality [5]	↓Sleep efficiency (SE), ↑ sleep-onset latency (SOL) reported [189,190]	Eveningness/social jetlag associate with higher symptom burden [71]	CBT-I is an effective adjunct [205,206]
PTSD	Insomnia and trauma-related nightmares are criterion-level; frequent awakenings, nocturnal panic, and persistent hyperarousal are common [30,209]	↓Total sleep time, ↓ sleep efficiency, ↑ awakenings/fragmentation, ↓ SWS, ↑ REM density and REM fragmentation [35,209]	Elevated nocturnal sympathetic and HPA activity, blunted nighttime heart-rate decline, and altered diurnal cortisol rhythms consistent with sustained hyperarousal [199–202]	CBT-I improves insomnia/PTSD symptoms; consider prazosin for nightmares and BBTI or eszopiclone–SSRI augmentation if needed [218,219,220,222,208]
Psychosis	Insomnia, fragmentation; high poor-sleep prevalence [225,226]	↓SE, ↑ SOL/WASO; spindle deficits in chronic stages [224–226,231]	Irregular rest-activity rhythms reported [229]	Sleep relates to symptom burden and cognition [31,226,228]
Substance use disorders	Insomnia frequent in active use and early recovery [36]	AUD: ↑ SOL, ↓ SE, ↓ SWS, ↑ REM density [266]	Instability during withdrawal/early abstinence [36,272]	Targeted sleep care supports engagement and lowers relapse risk [32,290]
ADHD/ ASD	Sleep-onset delay, short sleep; DSPS common [242,249]	ADHD adults: ↑ SWS despite insomnia complaints [243]; ASD: objective sleep problems scarce [245]	Delayed/irregular rhythms prevalent [248,249]	Stimulant timing impacts sleep; melatonin-pathway findings in ASD [37,38,257,262]
Obsessive-Compulsive Disorder	Insomnia, prolonged latency; evening rituals/ rumination [299]	↓SE; hyperarousal patterns in PSG/actigraphy [300]	Irregularity reported in subsets [300]	Coordinate CBT-I with ERP; adjust SSRI timing/ augmentation if needed [57]
Eating disorders	Insomnia & circadian delay; night-eating (other specified feeding or eating disorder) [30]	Fragmentation; variable SWS/REM in AN [301]	Eveningness/ irregularity described [303]	Sleep complaints link to mood/ metabolic risk [303]

### 6.1. Future directions

Sleep is both a modifiable risk factor and a mechanistic probe across diagnoses. Priorities include causal designs, explicit engagement, validated sensing and analytics, and translation into early-warning and treatment workflows.

### 6.2. Close the causality gap

Research should move beyond association to experimental manipulation of sleep with prespecified mediators and target-engagement readouts, so that changes in sleep can be linked causally to changes in psychiatric outcomes. High-frequency within-person longitudinal cohorts, interventional trials with formal mediation, adaptive and factorial designs, and well-designed single-case studies can all contribute. Quasi-experimental opportunities and genetic causal inference can complement trials. Preregistration, directed acyclic graphs, and routine reporting of effect sizes and target-engagement metrics should be standard.

### 6.3. Mechanistic neuroimaging and biomarkers

Trials should link sleep change to brain and cellular targets using multimodal assessment. Useful approaches include neuroimaging paired with electrophysiology, endocrine and circadian measures, and selected inflammatory and omics panels. Where appropriate, closed-loop perturbations of slow-wave or rapid eye movement sleep can serve as causal probes. Target engagement (for example, increased spindle density or alignment of circadian phase) should be specified a priori as the pathway to clinical change.

### 6.4. Artificial intelligence (AI) and sensors for objective sleep-psychiatry

Wearables and smartphones can provide continuous, low-burden data, but validation and clinical utility must precede deployment. Device algorithms should be evaluated against polysomnography across diagnostic groups, audited for bias, and documented transparently. Open data standards and preregistered analysis plans are recommended. Prospective studies should test whether risk signals change clinical decisions and improve outcomes. Integration with electronic health records should follow privacy-by-design principles and include human oversight.

### 6.5. Sleep as early-warning signal and treatment target

Clinical pathways should convert sleep metrics into action, with disorder-specific thresholds that trigger stepped care and timely behavioral or chronobiological supports, alongside universal screening, clear referral routes, deprescribing plans, clinician education, and equity considerations across settings and the lifespan. To improve comparability across studies, the field could converge on a concise core outcome set centered on high-yield, interpretable metrics such as sleep efficiency, a rapid eye movement fragmentation index, change in Insomnia Severity Index, circadian phase by dim-light melatonin onset, and a brief inflammatory panel (interleukin-6 and C-reactive protein).

## 7. Conclusion

Across diagnoses, sleep disturbance is neither incidental nor epiphenomenal: it interacts bidirectionally with core psychopathology and shares convergent substrates in prefrontal-amygdala circuits, thalamocortical oscillations, neuromodulatory systems (GABA, serotonin, dopamine, orexin), circadian timing, and stress-immune pathways. This common architecture helps explain why insomnia, hypersomnia, and circadian misalignment predict onsets, exacerbate symptom severity, and foreshadow relapse across mood, anxiety/trauma, psychotic, neurodevelopmental, and substance use disorders.

Clinically, sleep is a tractable lever. Routine, structured assessment (e.g., insomnia, circadian timing, apnea risk, nightmares) should be embedded in psychiatric care, with first-line behavioral/circadian strategies (CBT-I, light/chronotherapy, social-rhythm regularization) prioritized and pharmacologic options used judiciously, time-limited, and condition-matched. Although CBT-I is consistently identified as the gold standard for chronic insomnia, its reach remains far below clinical need: the high population prevalence of insomnia contrasts sharply with the limited availability of trained CBT-I clinicians and practical barriers related to cost, time, and access. As highlighted repeatedly in the insomnia literature, scaling CBT-I will require broader dissemination pathways, including brief formats, group-based approaches, digital programs, and stepped-care models, to ensure that evidence-based insomnia treatment is accessible at the population level. Objective monitoring (actigraphy, validated wearables) can augment history, individualize targets (efficiency, timing variability, REM fragmentation), and support stepped-care and relapse-prevention pathways. In parallel, research should move beyond association to mechanism-anchored intervention trials that demonstrate target engagement (e.g., spindles, DLMO, cortisol slope, inflammatory markers) and test whether changing sleep causally improves psychiatric outcomes.

Positioning sleep as both barometer and lever—measured continuously, interpreted mechanistically, and targeted proactively—offers a pragmatic route to earlier intervention, fewer relapses, and better functioning. Closing the causality gap, standardizing sensing/AI, and integrating sleep workflows into routine psychiatry are immediate priorities for the next decade.
